# Real Time Cascade Impactor Based On Surface Acoustic Wave Delay Lines for PM10 and PM2.5 Mass Concentration Measurement

**DOI:** 10.3390/s18010255

**Published:** 2018-01-16

**Authors:** Lyes Djoumi, Meddy Vanotti, Virginie Blondeau-Patissier

**Affiliations:** Franche-Comté Electronics Mechanics Thermal Science and Optics – Sciences and Technologies Institute (FEMTO-ST), Time and Frequency Department, 26, Chemin de l’Epitaphe, 25030 Besançon, France; meddy.vanotti@femto-st.fr (M.V.); virginie.blondeau@femto-st.fr (V.B.-P.)

**Keywords:** Surface Acoutic Waves, Love waves, delay lines, PM Sensor, PM10, PM2.5, cascade impactor, fine particles

## Abstract

In this research, Surface Acoustic Wave (SAW) sensors are combined with a cascade impactor to perform real time PM10 and PM2.5 mass concentration measurements. The SAW sensors consist of 125 MHz delay lines based on Love waves propagating on an AT-cut quartz substrate. The Love waves are guided on the substrate’s surface using a silica layer. SAW sensors themselves are not capable to discriminate particles by their size, therefore, particle separation based on aerodynamic diameter is achieved using a 3 Lpm dedicated cascade impactor. The latter was designed to integrate the SAW sensors which are monitored using a phase shift measurement. The collected particles impact on the acoustic sensor’s surface inducing a gravimetric effect that modifies the acoustic wave propagation conditions. The resulted phase shift allows the measurement of the mass deposited on the sensitive zone. The novel cascade impactor with SAW sensors as particle collecting stages is exposed to different aerosols in the 0–150 μg/m^3^ concentration range and proved to be able to detect and differentiate particles based on their size in real time. The system’s response was compared to a commercial optical counter based on light scattering technology and was found to be in good agreement with it.

## 1. Introduction

During the last few years, environmental pollution has become a big concern. In particular, fine particulate matter (PM) often reaches dangerous levels in the atmosphere of big cities around the world. Particulate matter having an aerodynamic diameter (AED) smaller than 10 μm and 2.5 μm, respectively PM10 and PM2.5, are a major threat on human health as they can easily penetrate the human lungs. These particles can cause cardiovascular diseases leading to death for extreme, cases [1,2,3,4]. Particle pollution is commonly described in μg/m^3^ for mass concentration. A number of instruments of various technologies can be used to measure it. The current trend is going toward high reliability, low cost and low maintenance instruments allowing access to data in real time. This permits the use of a large number of instruments simultaneously and to therefore obtain large geographical coverage [5,6,7].

Optical counters are very widely used for real time aerosol analysis due to their ability to deliver particle-size distribution over a wide range. However, the detection principle being based on light scattering, optical counters measure the optical diameter rather than the aerodynamic diameter. Consequently, these instruments need to be calibrated for each targeted particle type. This makes them poorly adapted for atmospheric pollution as it contains particles of various natures [8,9]. Moreover, optical methods are based on particle counting and hence do not directly provide mass concentration which also depends on the chemical composition of the particles [10]. Gravimetric methods are better suited to perform particle mass concentration measurement. However, the process of first separating the particles based on inertial forces and then weighting them is time consuming and often requires an operator. This compromises the possibility of real time measurement. Recent advancements on mass concentration measurement mainly concern miniaturization allowing the design of chip scale devices [11,12,13,14]. Instruments based on a gravimetric principle may require the use of complex electronic circuitry such as high frequency signal generation and acquisition. Compared to optical instruments, these issues may compromise miniaturization and cost of the entire system.

The sensing capabilities of Surface Acoustic Wave (SAW) sensors were first presented by the end of the 1970s [15]. Since then, their interest has been growing due to their high gravimetric sensitivity, up to 450 cm^2^/g for a device operating at 200 MHz [16]. Compared to Bulk Acoustic Wave devices, such as the Quartz Crystal Micro-balance (QCM), the acoustic energy in SAW devices is confined in the surface leading to a higher sensitivity [17]. For fine particle mass measurements, SAW sensors were used for the first time by Bowers et al. and proved to be 250 times more sensitive than a QCM [18]. A theoretical study of PM2.5 detection using a SAW resonator was later published by Hao et al. [19]. More recently, a high frequency SAW device to measure particles was presented by Thomas et al. [20].

In this paper, we propose a new gravimetric and real-time approach using Surface Acoustic Wave delay lines integrated in a cascade impactor. The goal of our research is to take advantage of the SAW’s gravimetric sensitivity combined with the selectivity based on inertial forces of cascade impactors. The first section of this paper is dedicated to the design of the SAW sensors. Particle separation based on a cascade impactor will be discussed in the second section. The results obtained with the integrated system will be presented in the last section.

## 2. SAW Sensor

**Figure 1:**
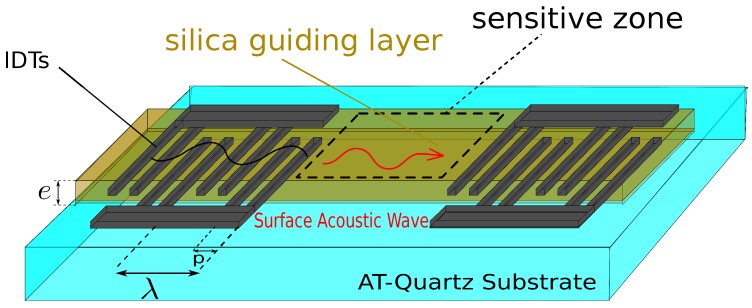
Structure of a Surface Acoustic Wave (SAW) delay line based on Love waves: p=10 μm, λ=40 μm.

Our SAW sensor is based on a delay line built on an AT-cut quartz substrate (cf. Figure 1). The choice of the AT-cut is based on the fact that it exhibits a low frequency-temperature coefficient (FTC) [21] making it well suited for sensor applications. The interdigitated electrodes (IDTs) consist of 200 nm thick aluminum double-electrodes deposited using a conventional photolithography process. Each finger is 5 μm wide and the space period *p* is 10 μm yielding to an acoustic wavelength λ=4×p = 40 μm (cf. Figure 1). The “double-electrode” configuration was chosen to get rid of triple transit reflection phenomenon, therefore avoiding the appearance of ripples in the interrogation bandwidth that compromises the phase measurement accuracy [22]. The sensor is based on Love waves (shear mode). The propagation of such a mode requires an additional layer on top of the substrate to guide the wave along the surface. This layer has to present a shear acoustic velocity $V_{s}$ lower than the substrate’s [23]. We used a silica guiding layer for this purpose ($V_{s\;\mathrm{silica}}=3764$ m/s).

The principle of a SAW delay line is shown in Figure 1 where an acoustic wave is generated on the surface of a piezoelectric substrate by applying an electric signal on interdigitated electrodes (IDTs). The acoustic wave propagates from one IDT to the other with a phase velocity that depends on the substrate and the physical properties of the guiding layer . The detection of particles is based on the gravimetric effect. Any modification on the surface, such as an additional mass, affects the conditions of propagation. The wave phase velocity decreases accordingly inducing an increase in the propagation delay.

**Figure 2:**
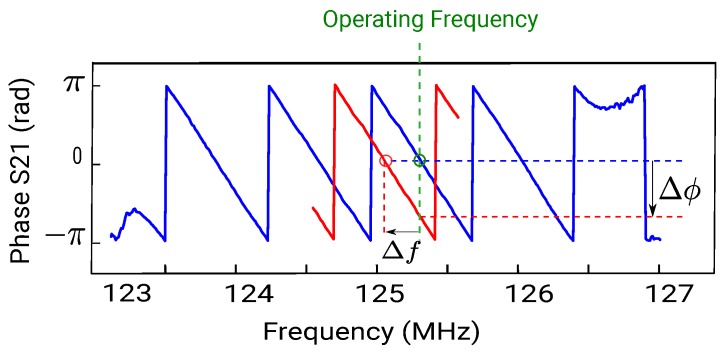
Phase monitoring at constant frequency: in blue the phase before the mass loading, in red the phase after the mass loading.

The acoustic shear wave velocity of the substrate being in the vicinity of 5100 m/s, the synchronous frequency of the device f=c/λ is 125 MHz as λ=40 μm. The wavelength is constant since it depends only on IDT geometry. Consequently, any variation of the wave phase velocity will linearly affect the synchronous frequency. This frequency decrease is measured by monitoring the phase signal with an open loop interrogation using a dedicated electronics [24]. Figure 2 presents the phase measurement principle at constant frequency. The blue curve represents the phase response of the SAW device before interacting with the particles. The red curve corresponds to the phase after the frequency shifts by Δf due to the mass effect. The phase being a linear function to frequency in the vicinity of the operating frequency (in green), the frequency shift can be determined by measuring the corresponding phase shift Δϕ.

The gravimetric sensitivity of a Love wave device *S*, expressed in cm^2^/g, depends on the energy density in the guiding layer. Therefore, the properties of the guide, such as its thickness, will directly affect the sensitivity of the device. In order to estimate the influence of the guiding layer geometry, we used software developed in our institute [25,26] to simulate the velocity of a Love wave propagating in a silica guide.

**Figure 3:**
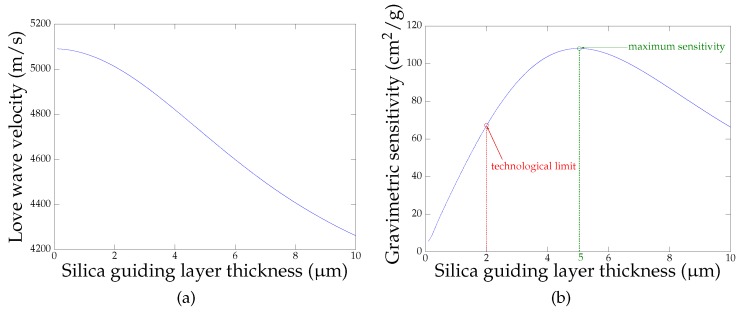
Love wave velocity (**a**) and the gravimetric sensitivity (**b**) as a function of the silica guide thickness.

Figure 3a shows the velocity of a Love wave as a function of the thickness of the silica guiding layer *e*. From this graph, one can calculate the gravimetric sensitivity of the device using the Equation (1) with $V_{0}=5100$ m/s the shear wave velocity of the substrate and ρ=2.2 g/cm^3^ the density of the silica guiding layer.

(1) $$S=\frac{\Delta V}{V_{0}}\cdot \frac{1}{\rho \times \Delta e}$$

**Figure 4:**
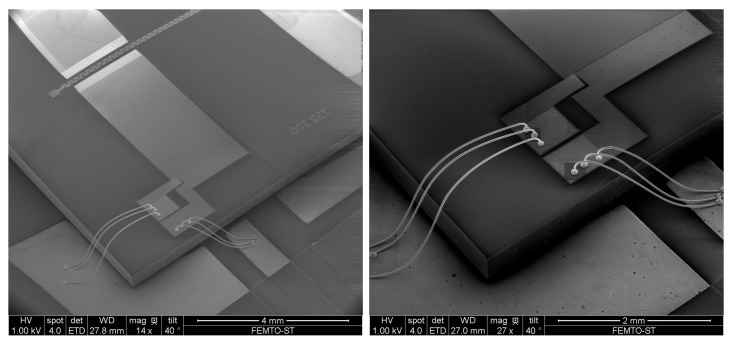
Scanning Electron Microscopy image of a Surface Acoustic Wave delay line mounted on a printed circuit board.

Figure 3b shows that the maximum sensitivity is obtained for a silica guide thickness of 5 μm. However, due to technological limitations, we fabricated Love devices with silica guiding layers comprised between 800 nm and 1.8 μm using Plasma Enhanced Chemical Vapor Deposition (PECVD). The chips were mounted on a printed circuit board using 25 μm gold wire bonds (cf. Figure 4).

**Figure 5:**
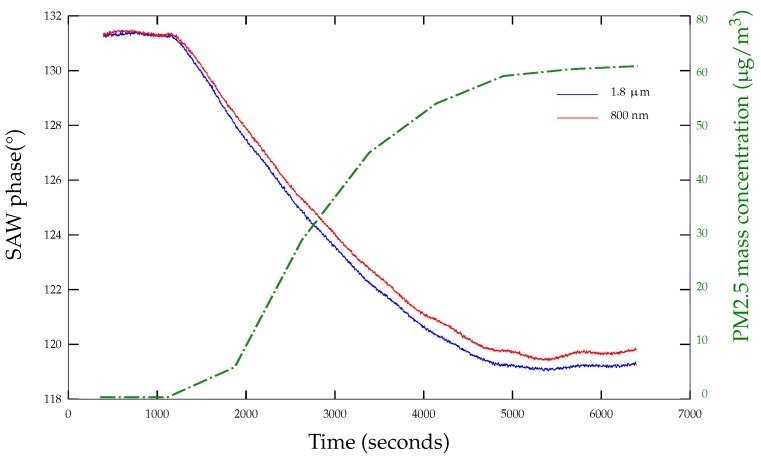
Love wave sensor with an 800 nm (red curve) and 1.8 μm (blue curve) silica guiding layer phase response to PM1 particles.

To compare their performances in practice, we exposed them to PM2.5 particles inside a temperature controlled Mikron VCE-1000. The particles are mainly submicron fine particles (PM1) generated from a burning candle. A reference aerosol spectrometer G 11-A (Grimm^®^, Ainring, Germany) based on light scattering is used to measure particle mass concentration in the room in real time. Figure 5 presents the phase of two Love wave devices exposed to particles inside the room. Both sensors see their phases decrease as the concentration of the particles in the room increases. However, one can observe that the SAW device with a 1.8 μm guiding layer shows neither a significantly greater phase shift, nor higher sensitivity compared to the one with a 800 nm thick guide. For this reason, we have chosen to work with 800 nm guides for our sensors since thickness homogeneity is more reliable for e<1 μm.

Although the previous experiment demonstrates the ability of Love wave delay lines to detect fine particles, the acoustic device itself cannot differentiate particles by size. To be able to target two different size ranges (PM2.5 and PM10), it is necessary to select the right particles before they are measured. In the next section, we describe how to achieve this particle separation.

## 3. Impactor Design

**Figure 6:**
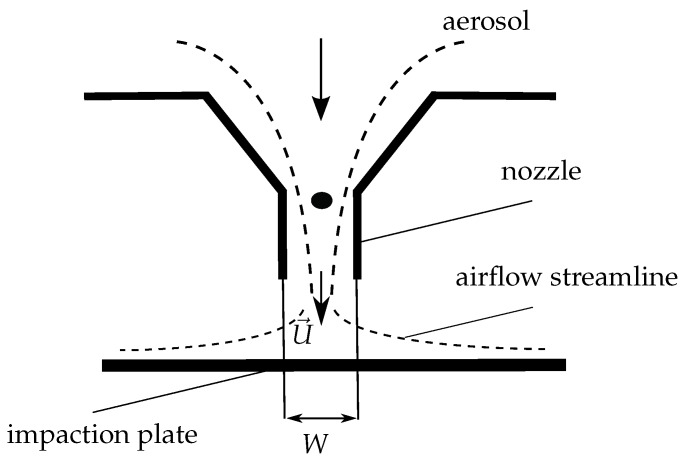
Impaction principle.

PM2.5 and PM10 are defined by their aerodynamic diameter; particle separation is therefore based on inertial forces [27]. Cascade impactors are very popular instruments for aerosol size characterization. These multistage inertial classifiers were introduced for the first time in 1945 [28] and have been widely used since then. In a cascade impactor, the aerosol is sampled at a constant flow rate. The air full of particles passes through nozzles that increase the flow velocity. As the airflow reaches the impaction plate, the particles with higher inertia deviate from the streamlines and impact on the plate to be collected (cf. Figure 6). Each impactor stage is characterized by a cut-off diameter noted $d_{50}$. The latter corresponds to 50% collection efficiency (analogous to the cut-off frequency at half power in electronics) and depends on the geometry of the device and the sampling flow rate.

In conventional impactors, the measurement cannot be performed in real time. Instead, impaction plates need to be removed and weighted before and after aerosol sampling in order to estimate the amount of particles present in each stage. As a consequence, one could only get an average measurement through the whole sampling time. Moreover, removing the impaction plates is time consuming and requires an operator. In our system, a SAW sensor is positioned on the impaction plate to measure the amount of particles in real time.

Whether a particle impacts on the impaction plate or not depends on the Stokes number which is linked to the particle aerodyanamic diameter $d_{p}$ through Equation (2) [29]

(2) $$S_{tk}=\frac{\rho_{p}d_{p}^{2}C_{c}U}{9\eta W},$$

where η is the air viscosity, *W* the nozzle diameter , $C_{c}$ the Cunningham slip factor, $\rho_{p}$ the particle density and *U* the average flow velocity in the nozzle. The cut-off diameter $d_{50}$ can be determined using Equation (3) where $Stk_{50}$ is the critical Stokes number corresponding to 50% efficiency. For round shape nozzles, we have $Stk_{50}≃0.24$ [30].

(3) $$d_{50}=\sqrt{\frac{9\eta WS_{tk50}}{\rho_{p}C_{c}U}}$$

**Figure 7:**
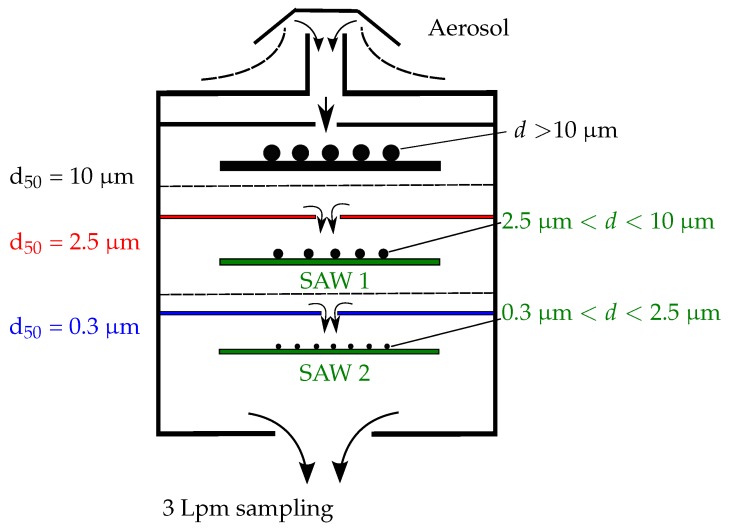
Schematic of a cascade impactor containing SAW sensors positioned as collection plates to measure the particle concentration in real time.

Using these equations, we designed a three stage impactor operating at 3 Lpm which contains two SAW sensors to perform real time analysis. Figure 7 presents a schematic layout of the device.

**Figure 8:**
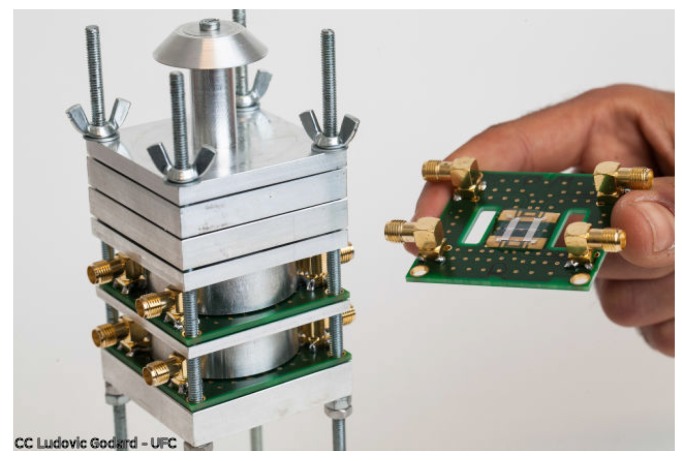
3 Lpm cascade impactor prototype with SAW sensors.

The top stage stops particles bigger than 10 μm. Since we target only smaller particles, these are collected on the impaction plate without being measured. The following two stages both contain SAW sensors of which the phase is monitored continuously at constant frequency (cf. Figure 2). The cut-off diameters of these stages are 2.5 μm and 0.3 μm. Consequently, the first sensor measures particles in the range [2.5 μm, 10 μm]. We label in this paper this stage as “PM10” even though it doesn’t measure all the particles smaller than 10 μm. The lower stage would be labeled “PM2.5”, collecting and measuring particles in the range [0.3 μm, 2.5 μm]. All the particles finer than 0.3 μm are neglected. This value was chosen to limit pressure drop in the impactor, hence reducing power consumption. It also corresponds to the detection limit of many commercial instruments. The nozzles are positioned directly above the sensitive zone of one delay line. This way, all the particles impact on it and none on the second delay line used as a reference. An aluminum prototype, fabricated in FEMTO-ST (Franche Comté Électronique Mécanique Thermique et Optique—Sciences et Techniques) institute, is shown in Figure 8. To avoid aerosol leakage, o-rings are positioned between all impactor stages. The described design has been patented [31].

## 4. Results and Discussion

### 4.1. SAW Phase Response to Particles

**Figure 9:**
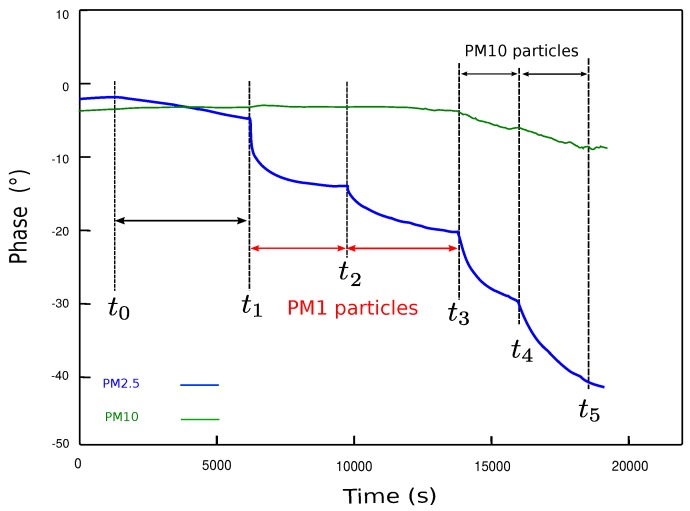
SAW sensor phase monitoring during successive exposures to particles in the [0, 10 μm] range: the PM2.5 stage (in blue) and the PM10 stage (in green).

Figure 9 presents the evolution of the SAW sensor’s phase during four successive exposures to particles in indoor conditions. The phase measurement is performed in a differential mode, which means that each sensor contains a couple of identical SAW delay lines: one receives the particles to measure and the other stays free of particles and works as a reference. By subtracting the two phase signals, we get rid of any contribution due to external operating conditions to measure only the gravimetric effect due to particles. During the first phase of the experiment from $t_{0}$ to $t_{1}$, no particles are generated. The blue curve, related to the PM2.5 stage, decreases very slightly indicating the presence of a few particles smaller than 2.5 μm in the room. At $t_{1}$, PM1 particles are generated using a burning candle. As a consequence, the phase of the PM2.5 stage decreases but not the PM10. Indeed, there are no particles bigger than 2.5 μm in the fume emanating from a burning candle [32]. The same PM1 particles are generated a second time at $t_{2}$ and the sensors respond in a similar fashion. At $t_{3}$, we expose the device to particles generated using a soldering iron. The emanating aerosol contains this time particles both in the ranges [0, 2.5 μm] and [2.5 μm, 10 μm] [33]. Therefore, both stages show a decreasing phase although the PM10 exhibits a weaker response compared to the PM2.5 indicating that fewer particles are larger than 2.5 μm [33]. At $t_{4}$ we repeat the same particle generation process with a similar response for both sensors, showing the repeatability of the measurement.

**Figure 10:**
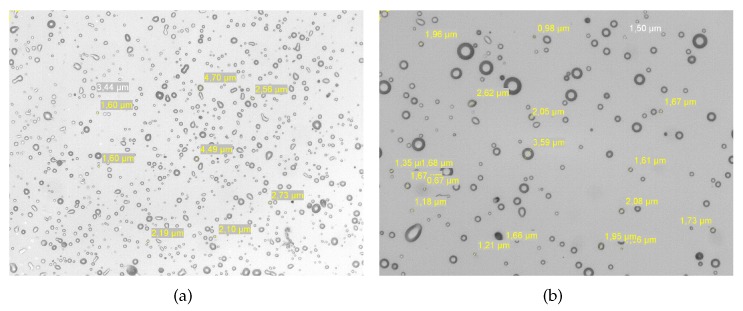
Image of the sensor’s surface on the PM2.5 stage: (**a**) zoom ×20 and (**b**) zoom ×50. The deposition (impaction) area corresponds to the nozzle geometry for this stage which is 4 nozzles of 0.38 mm diameter each.

**Figure 11:**
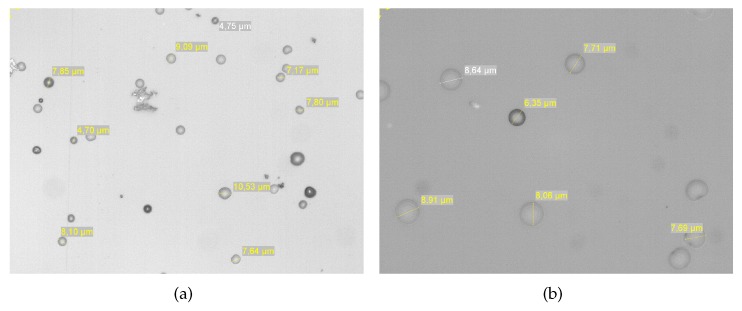
Image of the sensor’s surface on the PM10 stage: (**a**) zoom ×20 and (**b**) zoom ×50. The deposition (impaction) area corresponds to the nozzle geometry for this stage which is one nozzle of 2.19 mm diameter.

To make sure that each stage is actually measuring the targeted size range, we took images of the sensors surface at the end of the experiment using a microscope. On Figure 10 the surface of the PM2.5 stage and on Figure 11 the one of the PM10 stage. One can observe that, as expected, the PM2.5 contain more particles than the PM10 and that the particle sizes correspond well to the ranges [0.3 μm, 2.5 μm] and [2.5 μm, 10 μm]. It is worth noting that the distribution of particles on the sensitive surface is not homogeneous. It corresponds directly to the surface of the impactor nozzles.

### 4.2. Phase Variation vs. Particle Concentration

The gravimetric sensitivity of an acoustic wave sensor, deduced from the Sauerbrey formula [34], is given by Equation (4):

(4) $$S=\frac{df}{f_{0}}\cdot \frac{A}{dm},$$

with dm the mass variation, df the frequency shift, $f_{0}$ the operating frequency and *A* the surface of the sensitive zone. As we have seen above, the phase is linear with the frequency in the vicinity of the operating frequency (cf. Figure 2). Since the surface *A*, the frequency $f_{0}$ and the sensitivity *S* are all constant, the phase shift dϕ is proportional to the mass variation dm (dm∝dϕ). In our case, the mass of particles collected on the surface is m=c×v with *c* the particle concentration and v=Q×t the sampled aerosol volume. The mass variation would then be:

(5) $$dm=c\times Q\times dt\;\;\;\mathrm{and}\;\mathrm{therefore}\;\;\;\frac{dm}{dt}\propto c,$$

since the flow rate *Q* is constant over the whole sampling time and *c* can be considered constant as well during a very small period dt. Eventually, the particle concentration will be proportional to the phase variation in respect of time:

(6) $$c\propto \frac{d\phi }{dt}$$

**Figure 12:**
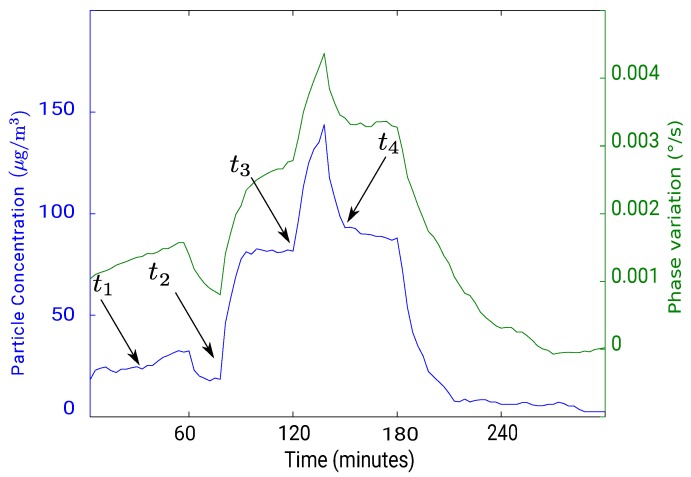
Comparison between PM2.5 SAW sensor phase derivative in respect of time $\frac{d\phi }{dt}$ (in green) and PM2.5 concentration measured with an optical counter (in blue).

**Figure 13:**
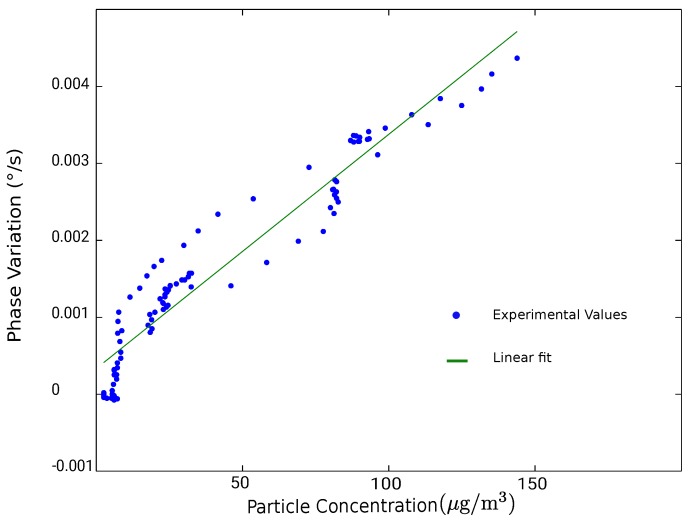
Phase variation as a function of the particle concentration.

Figure 12 presents a comparison between the phase variation $\frac{d\phi }{dt}$ (in green) of the SAW sensor with the concentration of the particles (in blue) measured with an optical counter “ePM” manufactured by Ecologicsense^®^ (Rousset, France). One phase measurement was recorded every second and the plotted data correspond to a sliding average over one hour window. We can observe that both curves follow a similar pattern indicating a correlation between the phase variation of the SAW sensor and the particle concentration. Figure 13 shows a plot of the phase variation as a function of the particle concentration from Figure 12. The system’s sensitivity, which is estimated by applying a linear fit of the plotted data, is 0.03 $\frac{m{}^{\circ}/s}{\mu g/m^{3}}$ meaning that with a phase measurement precision of 0.001°, we can detect 1 μg/m^3^ particle concentration within less than one minute sampling. 

## 5. Conclusions

In this research, we developed an original device for airborne particulate matter concentration monitoring. The device is based on surface acoustic waves sensors integrated within a dedicated cascade impactor. This new approach takes advantage of the SAW sensors intrinsic high sensitivity to gravimetric phenomena, combined with the selectivity of cascade impactors based on the aerodynamic diameter. Therefore, our device is better adapted for atmospheric pollution monitoring than optical counters. Compared with classical cascade impactors, it allows real time analysis as SAW sensors are positioned directly on the impaction plates to receive the particles constantly. Moreover, the differential configuration permits getting rid of operating conditions variations such as temperature and pressure. This is of great importance when performing outdoor measurements.
